# Supplementary material for: Magnetic domains and domain wall pinning in atomically thin CrBr3 revealed by nanoscale imaging
Source: Nat Commun. 2021 Mar 31;12:1989. doi: 10.1038/s41467-021-22239-4 (PMC8012586; doi:10.1038/s41467-021-22239-4)
Supplement: Supplementary file 1 — Supplementary Information [file 41467_2021_22239_MOESM1_ESM.pdf]

Supplementary Information: Magnetic domains and domain wall pinning in atomically thin $CrBr_3$ revealed by nanoscale imaging

Qi-Chao Sun[1,8*], Tiancheng Song[2,8], Eric Anderson[2], Andreas Brunner[1], Johannes Förster[3], Tetyana Shalomayeva[1], Takashi Taniguchi[4], Kenji Watanabe[4], Joachim Gräfe[3], Rainer Stöhr[1,5*], Xiaodong Xu[2,6] and Jörg Wrachtrup[1,7]

[1] *3. Physikalisches Institut, University of Stuttgart, 70569 Stuttgart, Germany*

[2] *Department of Physics, University of Washington, Seattle, Washington 98195, USA*

[3] *Max Planck Institute for Intelligent Systems, 70569 Stuttgart, Germany*

[4] *National Institute for Materials Science, Tsukuba, Ibaraki 305-0044, Japan*

[5] *Center for Applied Quantum Technology, University of Stuttgart, 70569 Stuttgart, Germany*

[6] *Department of Materials Science and Engineering, University of Washington, Seattle, Washington 98195, USA*

[7] *Max Planck Institute for Solid State Research, 70569 Stuttgart, Germany*

[8] *These authors contributed equally*

∗*E-mail: q.sun@pi3.uni-stuttgart.de; rainer.stoehr@pi3.uni-stuttgart.de*

1 Basics of scanning NV magnetometry

Experimental setup and measurement methods The schematics of our experimental setup is shown in Fig.1 (a). The sample and diamond tip are placed on two stacks of piezo-positioner units.

The sample substrate is glued on a Titanium sample holder which directly contacts with a thin copper plate (Fig.1 (b)). A heater and resistive thermometer is placed in the copper plate. The coplanar waveguides on the sample substrate and the print circuit board (PCB) are bonded with Au wires. The atomic force microscope works in a frequency modulation mode. The tuning fork is excited electrically and the readout signal is first amplified by using a home-made pre-amplifier placed at the microscope head, and then amplified by a voltage amplifier.

ODMR curve and sensitivity The hyperfine interactions between the ^{14}N nuclear spin (I = 1) of the NV center and the NV electronic spin results in three transitions, each separated by about 2.16 MHz in the $|m_s = 0\rangle$ to $|m_s = \pm 1\rangle$ transitions, as shown in Fig.1 (d). If the microwave applied to the NV center can drive the transition with Rabi frequency much larger than 2.16 MHz, the ODMR curve for each electronic spin transition shows a broad resonance and the hyperfine splitting lines can not be resolved. Fig.2 (a) shows a typical pulsed ODMR curve with a Rabi frequency of about 5 MHz. The shot-noise-limited sensitivity of an NV- magnetometer with the same microwave power can be calculated as $\eta = \frac{4}{3\sqrt{3}} \frac{w}{\gamma_e V \sqrt{R}}$, with fluorescence photon detection rate R, ODMR linewidth w and contrast V, and electronic spin gyromagnetic ratio $\gamma_e = 28$ GHzT^{-1}[1,2]. The prefactor $\frac{4}{3\sqrt{3}}$ originates from the steepest slope of the ODMR lineshape when assuming a Lorentzian resonance profile. This sensitivity, which is about 4.2 μTHz$^{-\frac{1}{2}}$ can be achieved when the microwave frequency detuning is set as $\frac{w}{2\sqrt{3}}$ from the resonance frequency. In order to achieve the optimal sensitivity, we reduce the microwave power to resolve the hyperfine splitting lines. In addition, we drive the three hyperfine splitting transitions simultaneously. Therefore, the ODMR curve obtained in this way shows five characteristic resonances lines as shown in Fig.2 (b). The

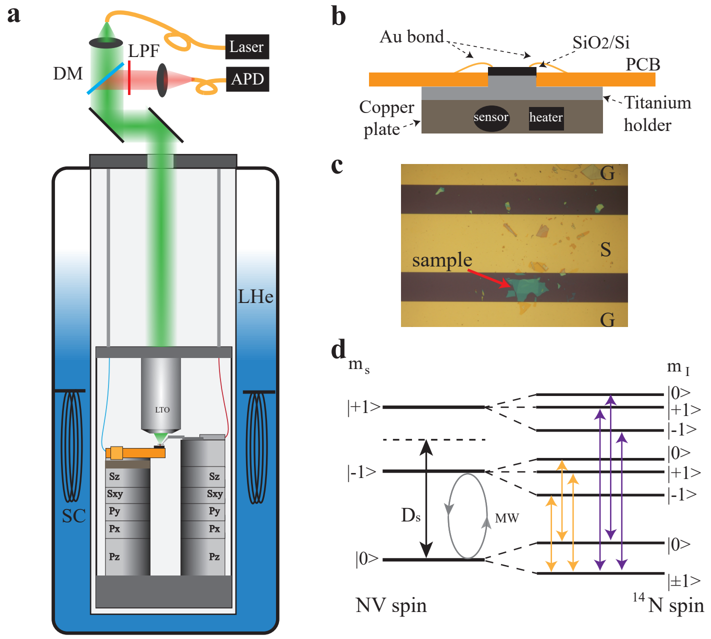

Figure 1: **The cryogenic scanning NV magnetometry.** **a**, Layout of the experimental setup. The liquid Helium (LHe) bath cryostat (attoLIQUID1000) has three pairs of superconducting coils (SC) which can generate vector magnetic field up to 0.5 T. A dichroic mirror (DM) and a band-pass filter are used to select the florescences photons of NV center, which are detected by a Si avalanche photodiode (APD). **b**, Schematic of the sample holder. **c**, Sample under test is transferred to one of the gaps of a coplanar waveguide. **d**, Energy levels of the ground state of negatively charged NV center, including the ^{14}N hyperfine splitting transitions.

central resonance line corresponds to the simultaneously excitation of the three hyperfine splitting transitions. The sensitivity calculated from this resonance line is about 0.29 $\mu\mathrm{THz}^{-\frac{1}{2}}$.

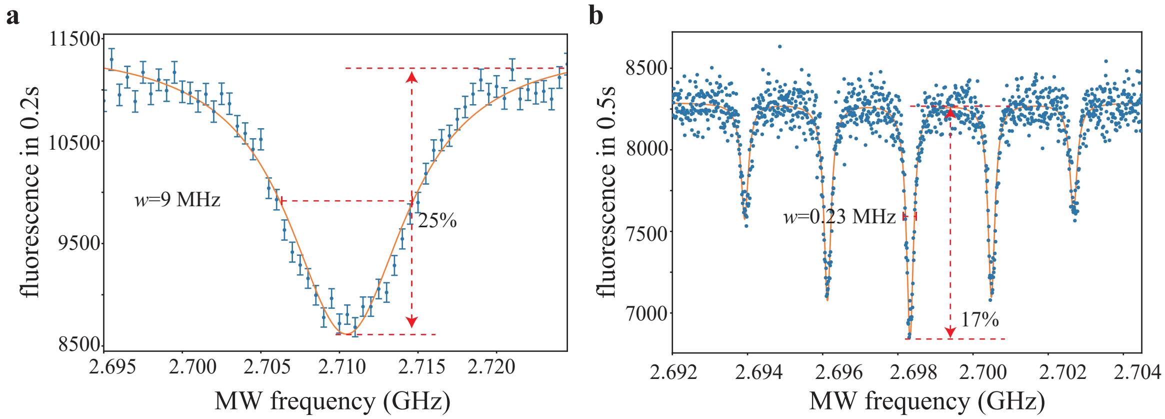

Figure 2: **Typical ODMR curves of NV centers in diamond tip.** **a**, Rabi frequency is about 5 MHz. **b**, Simultaneous excitation of the three hyperfine splitting transitions. The error bars in panel **a** represent 1 standard deviation, assuming Poisson statistics.

In the practical applications, we also need to consider the magnetic field measurement dynamic range, which should be in the order of 1 mT for few-layer $CrBr_3$. Therefore, most of the images are measured by recording the full ODMR spectrum by driving the NV transition with Rabi frequency of about 5 MHz. A few of the images shown in the Supplementary Information are measured by recording the NV fluorescence with the microwave frequency fixed at the steepest slope of the resonance line corresponding to the external field. With this so called iso-field method, one can achieve the optimal sensitivity, but the dynamic range is limited by the resonance linewidth.

Magnetization reconstruction from stray field map Although it is straightforward to calculate the stray magnetic field given the magnetization distribution, the inverse problem remains a chal-

lenge in magnetic source imaging due to the lack of a unique solution in most cases[3,4]. Here we discuss model of stray magnetic field generated by a 2D magnetic and show how to solve the inverse problem by introducing reasonable constraints. Fig.3 shows a 2D material in the xy-plane with coordinate $z = 0$ and the magnetization distribution $\mathbf{M}(x, y)$. The stray magnetic field in a parallel plane with a distance h from the the sample is given by

$$\mathbf{B}(x, y, h) = \int \mathrm{d}x'\mathrm{d}y'\mathbf{D}(x - x', y - y', h)\mathbf{M}(x', y'), \tag{1}$$

where $\mathbf{D}(x, y, z)$ is the dipolar tensor. With the convolution theorem, the integration in Eq. 1 can be rewritten as a product of the 2D Fourier transform of the corresponding quantities in the momentum space,

$$\tilde{\mathbf{B}}(k_x, k_y, h) = \tilde{\mathbf{D}}(k_x, k_y, h)\tilde{\mathbf{M}}(k_x, k_y). \tag{2}$$

The 2D Fourier transform of the components of the dipolar tensor reads as,

$$\tilde{\mathbf{D}}(k_x, k_y, h) = \frac{1}{2}\mu_0 e^{-kh} k \begin{bmatrix} -\frac{k_x^2}{k^2} & -\frac{k_x k_y}{k^2} & -i\frac{k_x}{k} \\ -\frac{k_x k_y}{k^2} & -\frac{k_y^2}{k^2} & -i\frac{k_y}{k} \\ -i\frac{k_x}{k} & -i\frac{k_y}{k} & 1 \end{bmatrix}, \tag{3}$$

if $k = \sqrt{k_x^2 + k_y^2} \neq 0$ and all components equal to zero if $k = 0$. The vanishing of non-zero components of matrix at zero momentum indicates that the uniform magnetization in a infinite large area has no contribution to the stray magnetic field.

Note that the three rows in the matrix in Eq. 3 are not independent. On the one hand, this means that in the momentum space, the three components of the vector stray magnetic field are not

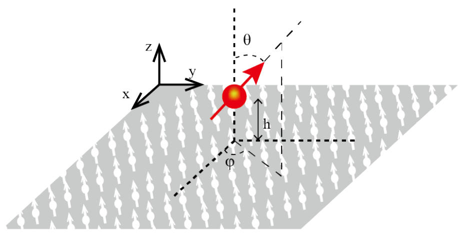

Figure 3: **Illustration of the reference frame used in the magnetization reconstruction.** The NV center and the spins of the 2D material are represented by the red ball and white balls with arrows indicating the orientation, respectively.

independent[5], e.g.,

$$\begin{aligned} \tilde{B}_x &= -i\frac{k_x}{k}\tilde{B}_z \\ \tilde{B}_y &= -i\frac{k_y}{k}\tilde{B}_z \quad (\text{for } k \neq 0) \end{aligned} \tag{4}$$

On the other hand, we cannot obtain a unique solution of magnetization given the stray magnetic fields in Eq.3 because the matrix is non-invertible. To obtain a unique solution, two additional constraints on the magnetization need to be introduced.

Here, we use the assumption of out-of-plane magnetization, that is $\mathbf{M}(x, y) = [0, 0, m_z(x, y)]^T$. With Eq.2 and Eq.3, the stray magnetic field component along z-axis is given by

$$\tilde{B}_z(k_x, k_y) = -\frac{i}{2}\mu_0 e^{-kh} k\tilde{m}_z(k_x, k_y). \tag{5}$$

The stray magnetic field component along the NV-axis can be related to the z component as

$$\tilde{B}_{NV}(k_x, k_y) = \cos(\phi)\sin(\theta)\tilde{B}_x(k_x, k_y) + \sin(\phi)\sin(\theta)\tilde{B}_y(k_x, k_y) + \cos(\theta)\tilde{B}_z(k_x, k_y). \tag{6}$$

The amplitude of magnetization $\tilde{m}_z(k_x, k_y)$ can be obtained by substituting Eq.4 and Eq.5 into Eq.6 and converted to the real space map by an inverse 2D Fourier transform. To reduce the reconstruction noise, a Hanning low-pass filter is used[6]. The direction of the NV axis is determined by adjusting the orientation of a 200 mT external magnetic field to maximumize the NV fluorescence[7]. The distance h can be obtained by fitting the magnetic field near the edges of the sample[8]. Besides the results shown in the main text, we also apply this method to the stray magnetic field image of a monolayer CrI_3 sample as shown in Fig.4. The average magnetization of this monolayer CrI_3 is about 16 $\mu_B \mathrm{nm}^{-2}$, which is consistent with that reported in Ref.6

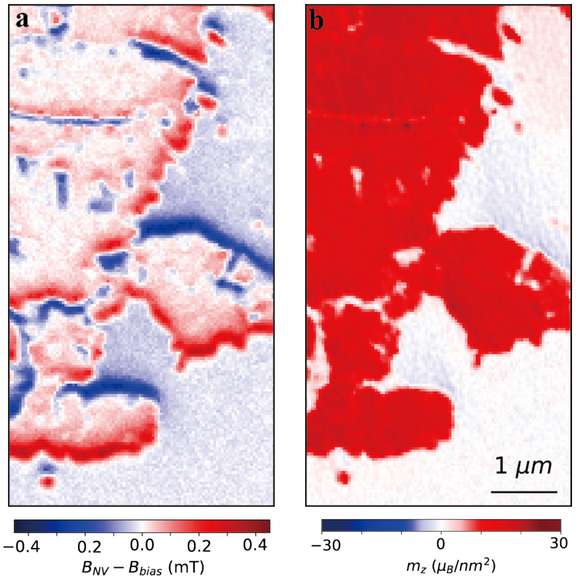

Figure 4: **Magnetization reconstruction of monolayer CrI_3.** **a**, Stray magnetic field of monolayer CrI_3 mapped by scanning NV magnetometer with external magnetic field of 0.2 T along the NV-axis. **b**, Magnetization image reconstructed from the stray magnetic field. The distance between the sample and NV center is about 80 nm in this measurement.

2 More information of the bilayer sample discussed in the main text

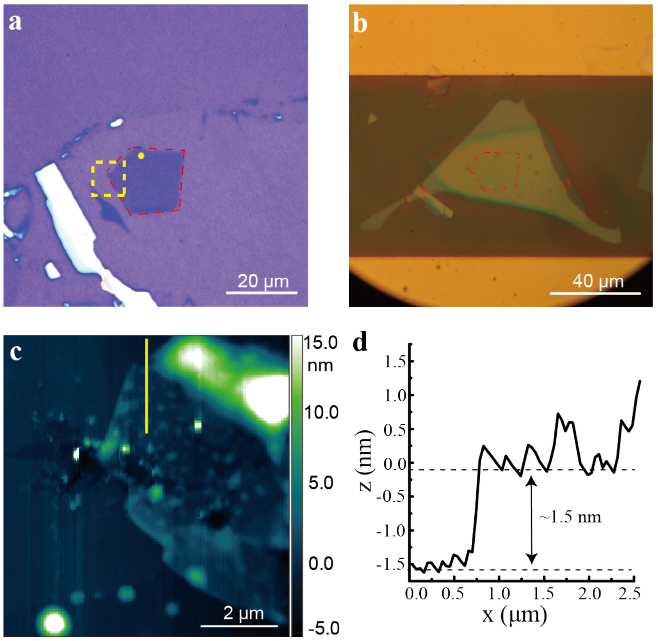

Figure 5: **Microscopy images of the sample.** **a** and **b**, Optical microscopy images of the bilayer $CrBr_3$ before and after being transferred to the gap of microwave coplanar waveguide, respectively. The red dashed boxes show the contour profile of the sample. **c**, Contact-mode AFM image of the area marked by the yellow dashed box in **a**. The magnetization images shown in Fig.2 and 3 of the main text and Fig.5 and 6 of the SI are obtained by scans in this area. **d**, AFM scan along the yellow line. The step height of 1.5 nm confirms that it is a bilayer sample. The magnetization images in Fig.4 of the main text and the Fig.7 of the SI is obtained in the area denoted by the solid circle in **a**.

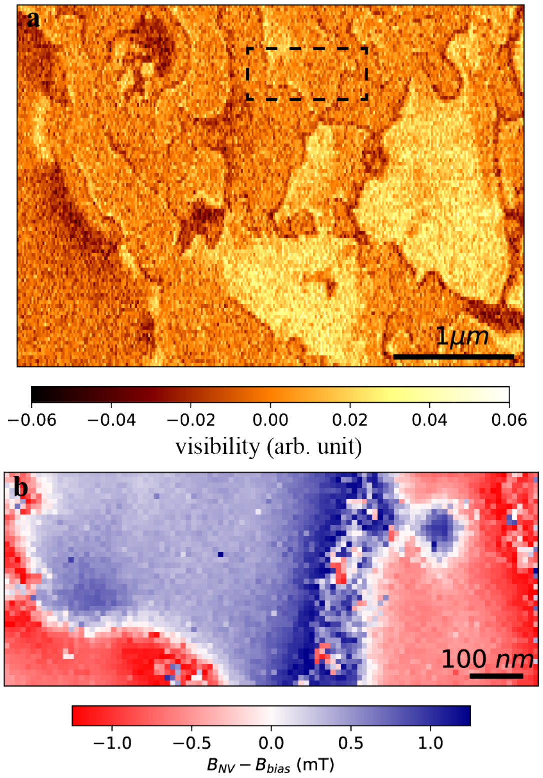

Figure 6: **Magnetic domains measured with another NV center.** **a**, Magnetic domains revealed in the dual-frequency iso-field image. **b**, Stray magnetic field image of the area marked by the dashed box in **a**. The magnetic domains wall in the two images are consistent with each other. The distance between the sample and NV center is about 60 nm in this measurement.

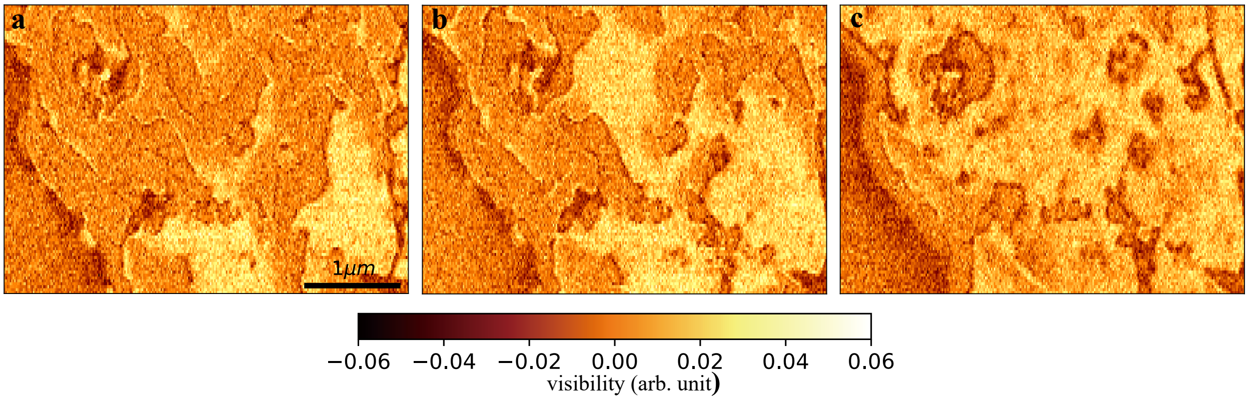

Figure 7: **Magnetic domain dynamics measured with the same NV center used in Fig.5 in this SI. a-c**, Dual-frequency iso-field images with external magnetic fields of 1, 3, and 10 mT, respectively.

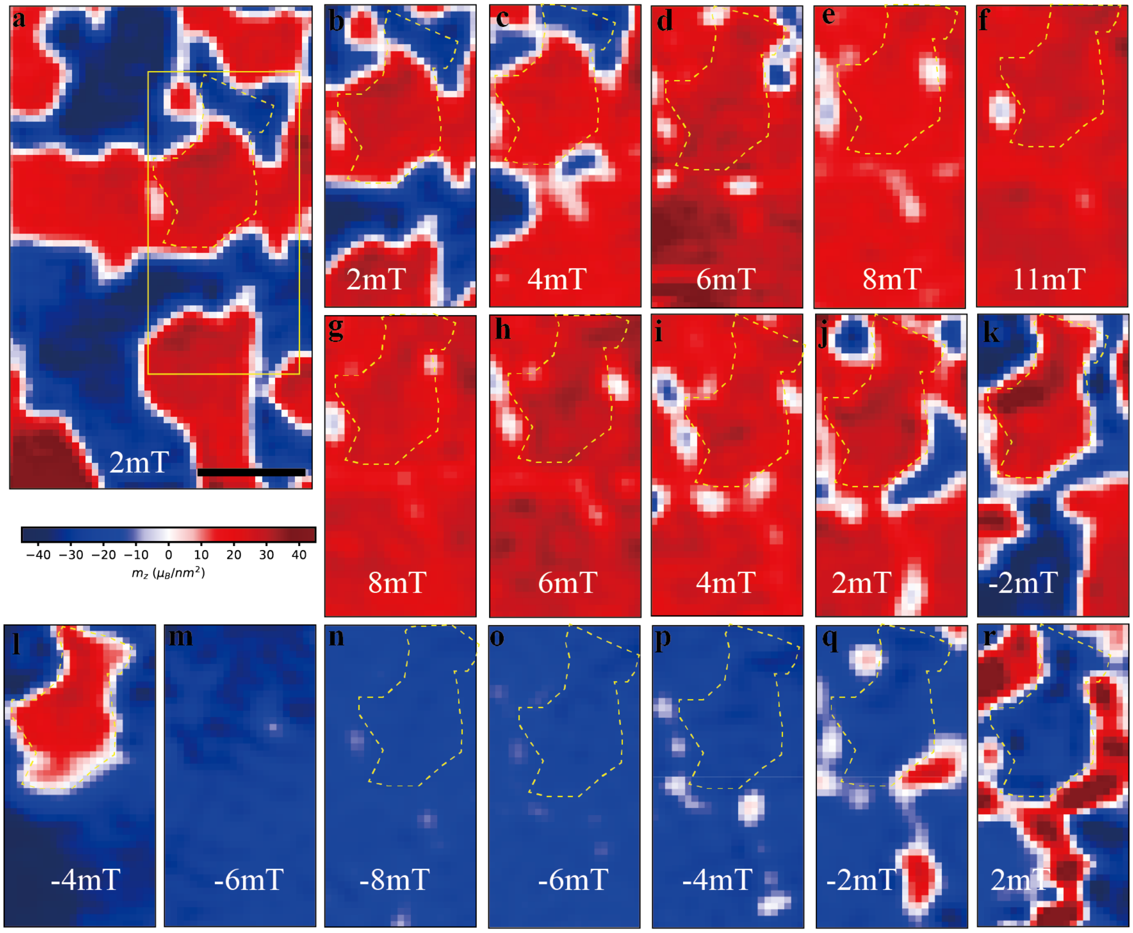

Figure 8: **Magnetization images used to calculate the hysteresis loop in Fig.4 of the main text.**

The scale bar stands for 1μm for all images.

3 Magnetic domains in 3L&4L $CrBr_3$ sample

Magnetic domain evolution This $CrBr_3$ sample has three-layer and four-layer areas, which are labeled in the AFM image (Fig.9). We measure the magnetic domain evolution in an area which has both 3L and 4L $CrBr_3$, as marked by the box #1 in Fig.9. The measurement with the thermally demagnetized sample and full magnetized sample are shown in Fig.10. The depinning field of the 3L $CrBr_3$ is much lower than the 4L $CrBr_3$. The domain reversal in 4L $CrBr_3$ is affected by domain wall pinning.

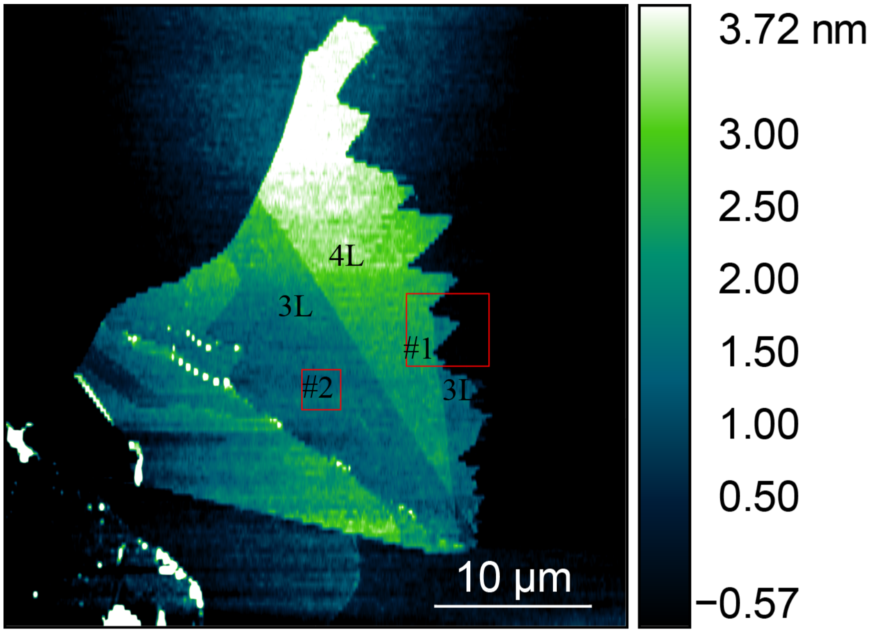

Figure 9: **AFM image of another sample.**

Discussion on laser heating effect. The magnetization at different laser power of a 3L $CrBr_3$ area is shown in Fig.11. We observe the magnetization starts to decrease when the laser power is higher than $\sim$30 μW. We also image the domain structure with laser power from 9 to 24 μW. No obvious domain motion has been observed in this measurement as shown in Fig. 12. All the other results

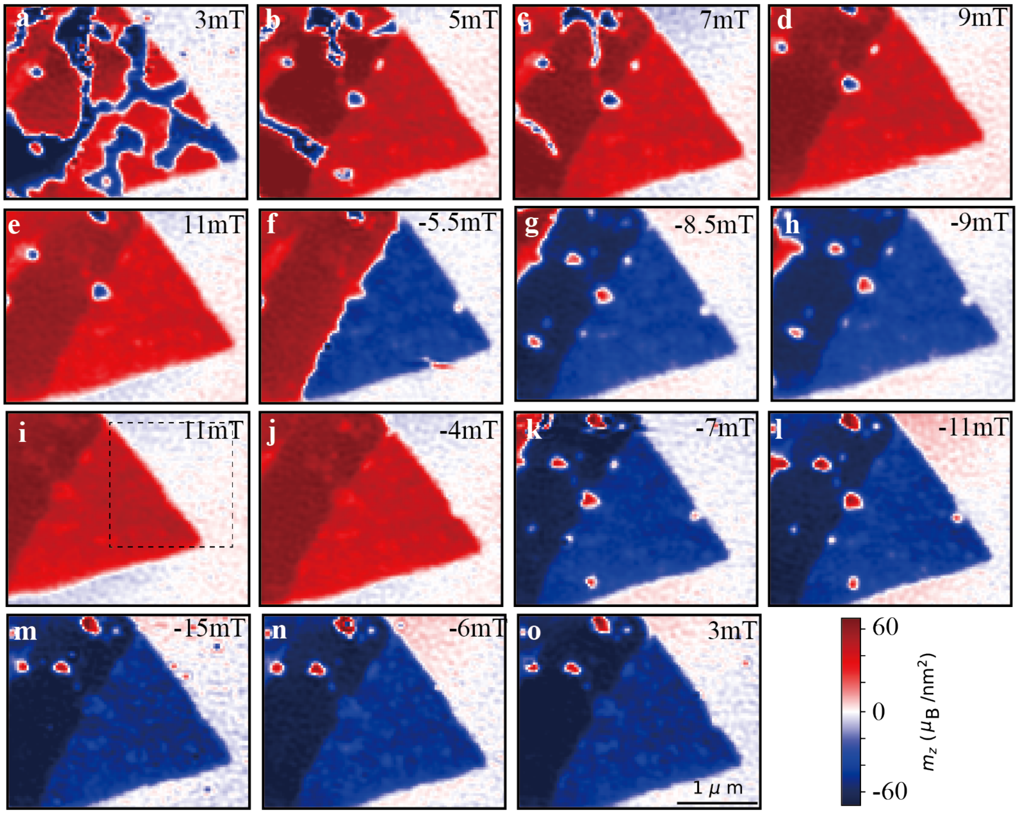

Figure 10: **Magnetic domain evolution upon varying the external magnetic field. a-h**, Magnetization of a thermally demagnetized sample upon varying the external field. **i-o**, The sample has been warmed up to 40 K and them cooled down to 4.2K at 0.5 T out-of-plane field.

shown in the main text and in the Supplementary Information are measured with laser power of about 15 μW. Therefore, the laser heating effect in these measurements is negligible.

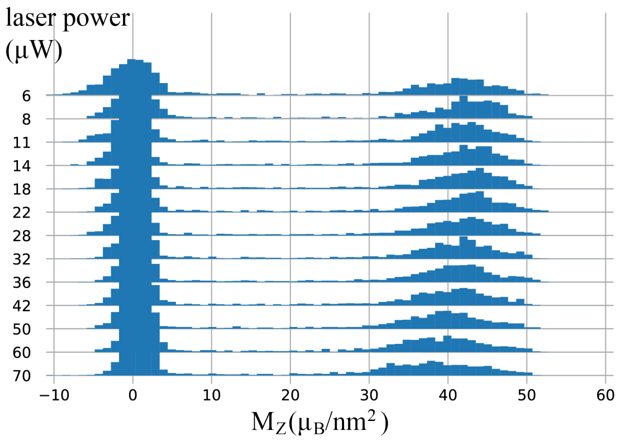

Figure 11: **Magnetization of 3L $CrBr_3$ upon increasing the laser power.** The histograms of the magnetization images taken at the area marked by the box in Fig.10 (i).

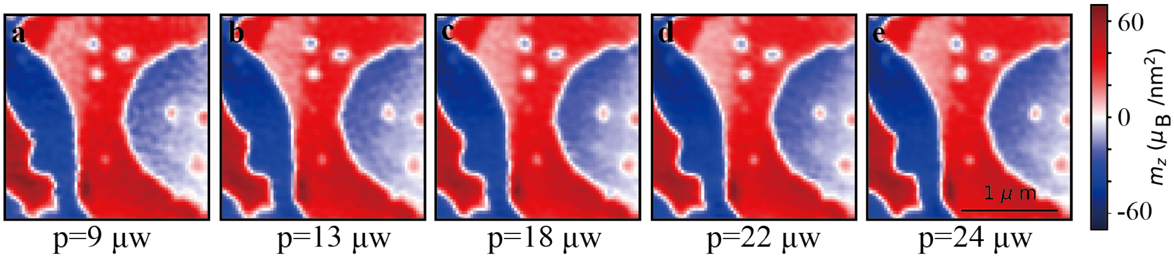

Figure 12: **Magnetic domain structure at various laser power.** The position of the measured area is marked by box #2 in Fig.9. The external magnetic field is 3 mT along NV axis.

4 Micromagnetic simulation

Simulations were carried out in a finite differences approach via MuMax3[9]. The simulation cells were set to a size of $1 \times 1 \times 1$ nm^3. The simulated system had an area of 3.0×1.5 μm^2 and a thickness of 2 nm, resulting in 2 layers of cubic cells. Periodic boundary conditions were set to one

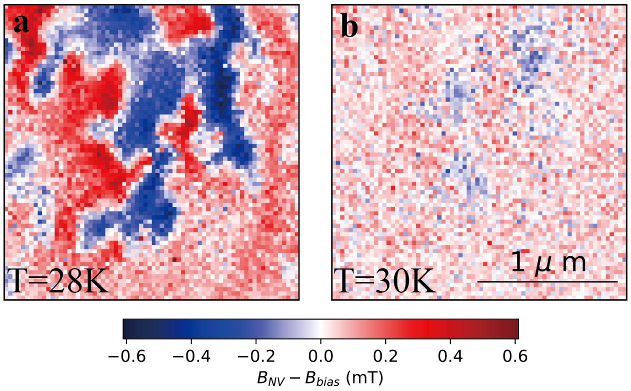

Figure 13: **Stray magnetic field of 3L $CrBr_3$ at temperatures close to the Curie temperature.** The external magnetic field is 3 mT along NV axis.

repletion in the lateral directions ["setPBC(1,1,0)"]. Temperature was neglected, so assumed to be 0 K. The samples shape was put in using the "ImageShape()" command. Material parameters are stated in the main text. The magnetic damping parameter was set to "alpha=1" for faster relaxation. Initial magnetization was set using "RandomMag()". The minimum energy state was determined using the "relax()" command.

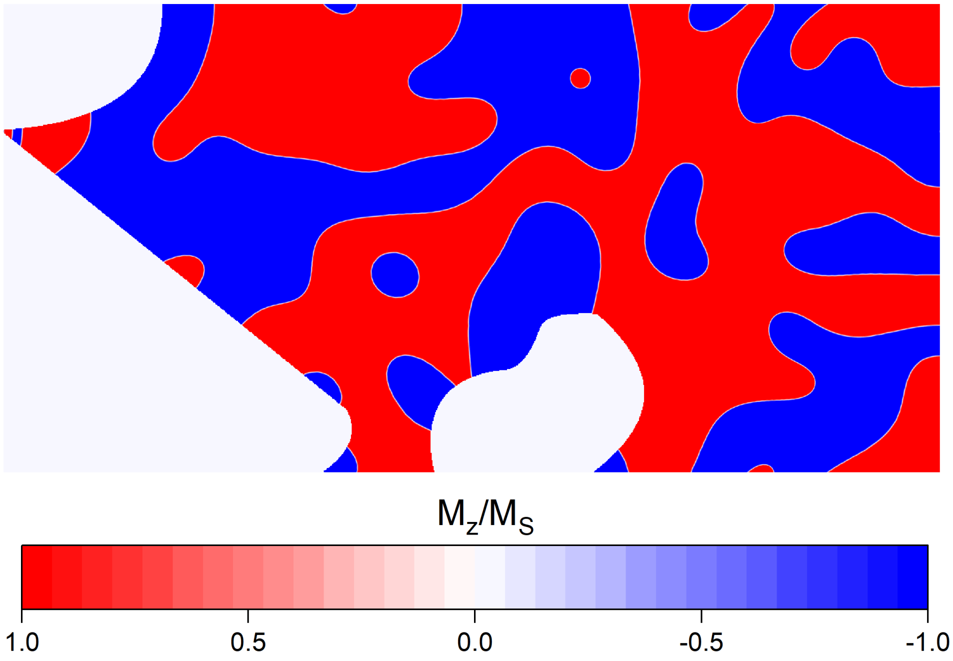

Figure 14: Simulation result of a system with a similar shape to the sample shown in Fig.2 of the main text.

1. Dréau, A. *et al.* Avoiding power broadening in optically detected magnetic resonance of single NV defects for enhanced dc magnetic field sensitivity. *Phys. Rev. B* **84** (2011).

2. Barry, J. F. *et al.* Sensitivity optimization for NV-diamond magnetometry. *Rev. Mod. Phys.* **92**, 608 (2020).

3. Dovzhenko, Y. *et al.* Magnetostatic twists in room-temperature skyrmions explored by nitrogen-vacancy center spin texture reconstruction. *Nat. Commun.* **9**, 2712 (2018).

4. Broadway, D. A. *et al.* Improved Current Density and Magnetization Reconstruction Through Vector Magnetic Field Measurements. *Phys. Rev. Applied* **14** (2020).

5. Lima, E. A. & Weiss, B. P. Obtaining vector magnetic field maps from single-component measurements of geological samples. *J. Geophys. Res.* **114**, 631 (2009).

6. Thiel, L. *et al.* Probing magnetism in 2D materials at the nanoscale with single-spin microscopy. *Science* **364**, 973–976 (2019).

7. Tetienne, J. P. *et al.* Magnetic-field-dependent photodynamics of single NV defects in diamond: an application to qualitative all-optical magnetic imaging. *New J. Phys.* **14**, 103033 (2012).

8. Tetienne, J.-P. *et al.* The nature of domain walls in ultrathin ferromagnets revealed by scanning nanomagnetometry. *Nat. Commun.* **6**, 6733 (2015).

9. Vansteenkiste, A. *et al.* The design and verification of MuMax3. *AIP Advances* **4**, 107133 (2014).
